# Supplementary material for: Incomplete vesicular docking limits synaptic strength under high release probability conditions
Source: eLife. 2020 Mar 31;9:e52137. doi: 10.7554/eLife.52137 (PMC7136020; doi:10.7554/eLife.52137)
Supplement: Source data 1. [file elife-52137-data1.zip › Appendix/Annotations on Figures.docx]

**Figure 1**

**Part A**

3 traces from experiment apr1515a (waves apr1515a_4_4, apr1515a_4_9, apr1515a_4_17).

**Part B**

Matrices of SV numbers.

Upper panels: imagewaves aug1816a_3_CT15 (control, 1.5 mM Ca) and aug1816a_18_TEA15 (1 mM TEA), from experiment aug1816a.

Middle panel: imagewave nov0917b_22_1-24_corr (5 mM TEA), from experiment nov0917b.

Lower panels: imagewaves feb0216_14_CT3 (control, 3 mM Ca) and feb0216_29_TEA3 (1 mM TEA), from experiment feb0216.

**Part C**

Upper panel:

Wave Var_CNTRL as a function of Mean_CNTRL, with corresponding sem values.

Second panel:

Wave Var_TEA as a function of Mean_TEA, with corresponding sem values.

Third panel:

Wave Var_5TEA as a function of Mean_5TEA, with corresponding sem values.

Fourth panel:

Wave Var_CNTRL3 as a function of Mean_CNTRL3, with corresponding sem values.

Fifth panel:

Wave Var_TEA3 as a function of Mean_TEA3, with corresponding sem values.

**Part D**

Waves depicting mean SV numbers across experiments as a function of stimulus number, in various experimental conditions. Waves are:

Mean_CNTRL (control, 1.5 mM Ca), Mean_TEA (1.5 mM Ca, 1 mM TEA), Mean_5TEA (1.5 mM Ca, 5 mM TEA), Mean_CNTRL3 (control, 3 mM Ca), Mean_TEA3 (3 mM Ca, 1 mM TEA). Each mean value is associated with the corresponding sem error bar.

**Part E**

Waves depicting mean P1 values for individual experiments:

P1st_CNTRLwTEA (control, 1.5 Ca) and P1st_TEA (after adding 1 mM TEA)

P1st_CNTRLw5TEA (control, 1.5 Ca) and P1st_5TEAwCT (after adding 5 mM TEA)

P1st_CNTRLwTEA3 (control, 3 Ca) and P1st_TEAwCT3 (after adding 1 mM TEA).

Overall means and corresponding error bars are also plotted.

**Part F**

Waves depicting mean Pmax values for individual experiments:

MaxP_CNTRLwTEA (control, 1.5 Ca) and MaxP_TEA (after adding 1 mM TEA)

MaxP_CNTRLw5TEA (control, 1.5 Ca) and MaxP_5TEAwCT (after adding 5 mM TEA)

MaxP_CNTRLwTEA3 (control, 3 Ca) and MaxP_TEAwCT3 (after adding 1 mM TEA).

Overall means and corresponding error bars are also plotted.

**Part G**

Waves depicting mean N values for individual experiments:

N1_CNTRL (control, 1.5 Ca)

N1_TEA (1.5 Ca, 1 mM TEA)

N1_5TEA (1.5 Ca, 5 mM TEA)

N1_CNTRL3 (control, 3 Ca)

N1_TEA3 (3 Ca, 1 mM TEA)

Overall means and corresponding error bars are also plotted.

**Part H**

Waves depicting mean PPR values for individual experiments:

PPR_CNTRL (control, 1.5 Ca)

PPR _TEA (1.5 Ca, 1 mM TEA)

PPR _5TEA (1.5 Ca, 5 mM TEA)

PPR _CNTRL3 (control, 3 Ca)

PPR _TEA3 (3 Ca, 1 mM TEA)

Overall means and corresponding error bars are also plotted.

**Figure 2**

**Part A**

Left: Wave 2_Var_Vesi plotted as a function of wave 2_Mean_vesi, showing the result of variance mean analysis with SV counting.

Center: Wave 2_Var plotted as a function of wave 2_Mean, showing the result of variance mean analysis with peak EPSC amplitudes.

Right: Wave 2_VarCorr plotted as a function of wave 2_Mean, showing the result of variance mean analysis with peak EPSC amplitudes, after correction for quantal amplitude variance.

**Part B**

Wave P1stFCT_IndivCorr, listing pairs of P1 values (using SV counting or EPSC peak amplitudes with correction) for 6 experiments. Corresponding means and sem values are also shown.

**Part C**

Wave PmaxFCT_IndivCorr, listing pairs of Pmax values (using SV counting or EPSC peak amplitudes with correction) for 6 experiments. Corresponding means and sem values are also shown.

**Part D**

Wave N_CT_indiv, listing pairs of N values (using SV counting or EPSC peak amplitudes with correction) for 6 experiments. Corresponding means and sem values are also shown.

**Part E**

Wave Q_indiv_CTCorr, listing pairs of q values (using SV counting or EPSC peak amplitudes with correction) for 6 experiments. Corresponding means and sem values are also shown.

**Figure 3**

**Part A**

First line: Calcium images fb0717a8_imgw32Corr (control), fb0717a8_imgw42Corr (peak calcium response) in 1.5 Ca

Second line: Calcium images fb0717a17_imgw27Corr (control), fb0717a17_imgw42Corr (peak calcium response) in 1.5 Ca + 1 TEA

Third line: Calcium images mr0817b9_imgw28 (control), mr0817b9_imgw43 (peak calcium response) in 3 Ca

Fourth line: Calcium images mr0817b15_imgw2 (control), mr0817b15_imgw42 (peak calcium response) in 3 Ca + 1 TEA

**Part B**

Upper panel:

Wave DF, showing peak calcium values for the experiment fb0717a, the same as that illustrated in the upper panel of part A, as a function of time.

Lower panel:

Traces from experiment fb0717a, the same as that illustrated in the upper panel of part A. Traces a8 (before TEA), a13 (during TEA) and a21 (washout) are shown.

**Part C**

Upper panel:

Waves line_ct_1.5_avg (control) and line_tea_1.5_avg (1 mM TEA) depict mean peak calcium values as a function of AP number.

Lower panel:

Waves AP1_ct1.5 and AP1_tea1.5Wct:

From 5 experiments, peak DF/Fo starting in 1.5 mM Ca, and adding 1 mM TEA

Corresponding mean and sem values are respectively:

ct_1.5_avg and ct_1.5_sem

tea_1.5Wct_avg and tea_1.5Wct_sem

Waves AP1_tea1.5W5tea and AP1_5tea1.5:

From 4 experiments, peak DF/F0 starting in 1.5 mM Ca and 1 mM TEA, and adding 4 mM TEA.

Corresponding mean and sem values are respectively:

tea_1.5W5tea_avg and tea_1.5W5tea_sem

5tea_1.5_avg and 5tea_1.5_sem

Waves AP1_ct3 and AP1_tea3:

From 5 experiments, peak DF/F0 starting in 3 mM Ca, and adding 1 mM TEA.

Corresponding mean and sem values are respectively:

ct_3_avg and ct_3_sem

tea_3_avg and tea_3_sem

**Figure 4**

**Part A**

wave 3: Probablity of release as a function of calcium concentration (uncaging experiments), from the model of Miki et al. 2018.

**Part B**

wave 1: Probablity of release as a function of calcium concentration (AP stimulation), from the model of Miki et al. 2018.

**Part C**

Original wave 1, as well as two scaled versions of the same wave (scaling factors 0.47 and 0.22).

Average values of P1 values in various experimental conditions:

p1_15Ca_newVan: 1.5 mM Ca (control, 1 mM TEA and 5 mM TEA)

p1_3Ca1: 3 mM Ca (control and 1 mM TEA)

Associated sem error bars are also shown.

**Figure 1-Figure supplement 1**

This figure is self-explanatory.

**Figure 4-Figure supplement 1**

This figure is self-explanatory.
